# Natural Radiosensitizers in Radiotherapy: Cancer Treatment by Combining Ionizing Radiation with Resveratrol

**DOI:** 10.3390/ijms231810627

**Published:** 2022-09-13

**Authors:** Dominika Komorowska, Tomasz Radzik, Sebastian Kalenik, Aleksandra Rodacka

**Affiliations:** 1 Department of Molecular Biophysics, Faculty of Biology and Environmental Protection, University of Lodz, 141/143 Pomorska St., 90-236 Lodz, Poland; 2 MARINEX International, 4 Placowa St., 93-446 Lodz, Poland

**Keywords:** radiotherapy, radiosensitizer, ionizing radiation, free radicals, DNA damage, resveratrol, paclitaxel, curcumin, genistein, papaverine

## Abstract

Conventional cancer treatment is mainly based on the surgical removal of the tumor followed by radiotherapy and/or chemotherapy. When surgical removal is not possible, radiotherapy and, less often, chemotherapy is the only way to treat patients. However, despite significant progress in understanding the molecular mechanisms of carcinogenesis and developments in modern radiotherapy techniques, radiotherapy (alone or in combination) does not always guarantee treatment success. One of the main causes is the radioresistance of cancer cells. Increasing the radiosensitivity of cancer cells improves the processes leading to their elimination during radiotherapy and prolonging the survival of cancer patients. In order to enhance the effect of radiotherapy in the treatment of radioresistant neoplasms, radiosensitizers are used. In clinical practice, synthetic radiosensitizers are commonly applied, but scientists have recently focused on using natural products (phytocompounds) as adjuvants in radiotherapy. In this review article, we only discuss naturally occurring radiosensitizers currently in clinical trials (paclitaxel, curcumin, genistein, and papaverine) and those whose radiation sensitizing effects, such as resveratrol, have been repeatedly confirmed by many independent studies.

## 1. Introduction

The increase in life expectancy, development of industry, environmental pollution, and growing obesity rates contribute to increased incidences of cancer. In more developed countries, cancer incidence is higher than in less developed countries; however, due to less diagnosis and treatment, less developed countries lead in terms of mortality [1]. Conventional cancer treatment is mainly based on surgical removal of the tumor followed by radiotherapy (RT) and/or chemotherapy. When surgical removal of the tumor is not possible, radiotherapy, and less often chemotherapy, is the only way to treat patients. Although radiotherapy and chemotherapy have the same goals, there are key differences between the two types of therapy. The major difference is the way they are delivered. During chemotherapy, drugs are taken by mouth or injection and usually expose the whole body to cancer-fighting drugs, radiotherapy is usually a local treatment. This means that RT is usually targeted and only affects the part of the body that needs treatment. Radiotherapy is scheduled to damage cancer cells with as little damage as possible to nearby healthy cells. Chemotherapy and radiotherapy are sometimes used together to treat certain types of cancer. This treatment is called combination therapy and may be recommended if cancer cells cannot be surgically removed, are likely to spread to other areas of the body, or if the cancer is not responding to one particular type of treatment. However, despite significant progress in understanding the molecular mechanisms of carcinogenesis and the development of modern radiotherapy techniques, radiotherapy (alone or in combination) does not always guarantee treatment success. One of the main causes is the radioresistance of cancer cells. 

One of the processes responsible for the radioresistance of cancer cells is the effectiveness of DNA damage repair after exposure to radiation. According to the basic assumption of radiobiology, cells with a strong ability to activate DNA repair processes are more resistant to radiation compared to cells with a weaker repair capacity. The degree of radiation-induced DNA damage activates various signaling pathways that promote the apoptosis or survival of cancer cells. Studies have shown that the Wnt/β-catenin pathway, NF-κB pathway, Akt/cyclin D1/CDK4 survival signaling pathway, and autophagy are associated with radiological resistance in cancer [2].

Another process that influences the radioresistance of neoplastic cells is the activation of cell checkpoint pathways. Cells are often blocked in the G1/S or G2/M phase, which allows time for DNA repair. Following radiation exposure, cell cycle arrest mainly occurs in the G2/M phase, which may be followed by G1-phase arrest. It is known that cells in the S phase show generally greater radioresistance than cells in G1 and G2, and cells in M are very radiosensitive. Quiescent cells in a G0 state are also radioresistant [3]. One of the hallmarks of cancer cells is the activation of the epithelial-mesenchymal transition process (EMT) by which epithelial cells transform into mesenchymal cells and acquire migratory and invasive abilities. ETM is associated with enhancement resistance to radiotherapy and poor prognosis in multiple types of malignant tumors [4]. For many types of cancer, the main factor related to radioresistance is the presence of cancer stem cells (CSC) inside tumors, which are responsible for metastases, relapses, RT failure, and poor prognosis in cancer patients [5]. Changes in the tumor microenvironment may also influence the development of radioresistance cancer cells. Many studies have shown that hypoxic environments promote the transformation of tumor cell metabolism from oxidative metabolism to anaerobic glycolysis, which induces the development of radioresistance in tumor cells [6,7]. The role of hypoxia in enhancing radioresistance has been fully described by Kabakov and Yakimov [8]. The authors conclude that chronic or prolonged hypoxia is a complex process that includes alterations in gene expression, signaling pathways, epigenetic regulation, the work of chaperones, autophagy, secretory activity, and other stress-sensitive mechanisms of the involved cancer cells. Many of those alterations are cancer cell-adapting mechanisms that contribute to the enhanced radioresistance of hypoxic tumors [8]. 

As described above, the radioresistance of cancer cells is a complex process. Understanding these mechanisms is essential to identify novel therapeutic targets and signaling pathways whose inhibition/activation will increase radiosensitivity and improve cancer treatment. Some researchers believe that the sensitivity of the cancer cell itself is a major factor in determining the tumor’s response to radiation [9].

In order to increase the effect of radiotherapy in treating radioresistant neoplasms, radiosensitizers are used. In clinical practice, synthetic radiosensitizers are commonly applied, but scientists have recently focused on using natural products (phytocompounds) as adjuvants in radiotherapy [10,11,12,13,14,15]. The mechanisms of the radiosensitizer’s action are described in a separate chapter. The enormous potential of substances found in plants has been known for centuries [16,17]. It is estimated that 60–80% of antibiotics and cancer drugs derive directly from naturally occurring species [18,19]. Substances with potential medicinal use are still waiting to be discovered in hundreds of thousands of plants, fungi, and animals [20]. Owing to scientific research, some compounds of natural origin used in treatments may apply to new therapies [21]. One of many examples is papaverine, which until now has been used as a smooth muscle relaxant and is currently being investigated as a radiosensitizer in treating non-small cell lung cancer (NSCLC) or lung metastases [13]. Several compounds with plant origins are currently included in clinical trials as radiosensitizers (Table 1). In addition to the aforementioned papaverine, there is also curcumin, paclitaxel, and genistein. Resveratrol (RSV) is another compound that may improve the treatment of many cancer types by enhancing radiotherapy. Despite numerous in vitro studies, as well as several in vivo studies confirming the radiosensitizing properties of resveratrol, there are still no clinical studies in this aspect.

The purpose of this review is to present clinical trials using compounds of plant origin as a radiosensitizer. In addition, we review the literature on the radiosensitizing effects of resveratrol for many types of cancer cells. In addition, we reviewed data from clinical trials assessing the effects of resveratrol in the treatment of cancer and selected age-related diseases.

## 2. Radiotherapy

Cancer continues to be one of the greatest challenges for healthcare. According to a report prepared by the International Agency for Research on Cancer (IARC), the number of deaths is expected to exceed 13 million by 2030, and confirmed cases would oscillate around 22 million patients. In order to reduce the number of deaths due to cancer, various methods of treatment have been developed, including radiotherapy, chemotherapy, surgery, and immunotherapy [10]. Nevertheless, radiotherapy is still one of the most prevalent cancer treatment methods worldwide. Approximately 60% of patients receive curative radiotherapy in the USA, despite advances in other treatments in the 127 years since its invention [22]. This finding shows that RT has proved to be an invaluable method in oncological treatment. 

RT is expected to eliminate a specific tumor and directly hit cancer cells with an emitted ionizing radiation (IR). The latter process damages the cell nucleus, leading to the death of cancer cells and thus eliminating the tumor. Compared to conventional pharmacological treatment, where administered cytostatics have a detrimental effect on healthy tissues throughout the body, modern/state-of-the-art radiotherapy focuses on directing a high-energy beam of photons to a specific location to kill cancer cells, leading to a reduced tumor mass and thus minimizing the dose and toxicity to neighboring normal tissue [12]. It should also be emphasized that radiotherapy combined with surgery or chemotherapy significantly increases the effectiveness of oncological treatment.

**Figure 1:**
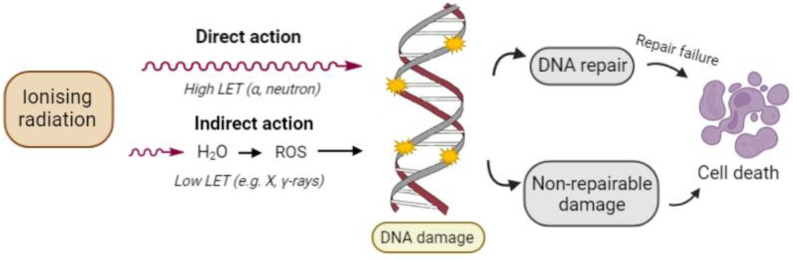
Direct and indirect action of ionizing radiation. In direct action—radiation directly interacts with DNA, causing DNA damage. In indirect action—radiation interacts with other molecules in cells, especially water molecules, producing reactive oxygen species (ROS), which induces DNA damage. DNA damage leads to cell death if not properly repaired.

Ionizing radiation may act directly or indirectly on cancer cell death mechanisms depending on where the IR energy is absorbed (Figure 1). When energy is deposited in a macromolecule associated with observable biological effects such as DNA and proteins, it is called a direct effect of radiation. It is the dominant process in the interaction of high linear energy transfer (LET) particles with biological materials. Alternatively, energy may be absorbed in an organism’s water because water molecules are the predominant molecule in living organisms (about 80% of the mass of a living cell is water). Practically a major proportion of radiation energy is absorbed by cellular water. Several complex chemical transformations occur in water under the influence of ionizing radiation. This process is called water radiolysis. About two-thirds of the biological damage by low LET radiation or sparse IR such as X-rays or electrons is due to indirect action. Evidence indicates that the biological effects of radiotherapy mainly derive from damage to DNA, a critical target of ionizing radiation in the human body. Cancer cells whose DNA is irreparably damaged stop dividing or die. When damaged cells die, they are broken down and eliminated by the body’s natural processes [23].

Unfortunately, despite innovative solutions in radiotherapy, which improve the therapeutic effect in patients, there are still complications that hinder oncological treatment, such as cancer stem cells (CSC), tumor heterogeneity, tumor recurrence, low cell radiosensitivity, or damage due to IR of healthy tissues [10]. Tumor heterogeneity influences the response variability of the tumor subpopulation to radiation, which reduces the effectiveness of the therapy. The main factor responsible for the radioresistance of neoplastic cells is neoplastic stem cells. RT resistance is associated with radiotherapy treatment failure, metastases, and relapses [5]. Today, treatment plans remain the same for each class of cancer. The molecular profile characteristic of each type of cancer is not considered, which would otherwise help increase the treatment’s effectiveness [12].

Clinical studies show that approximately 70% of patients require radiotherapy in oncological therapy. Moreover, in some cases, only radiation therapy remains the only form of treatment. There is a great need for research and development of various methods to improve the radiation sensitivity used in radiotherapy. Currently, research is being conducted on radiation-sensitizing compounds that can increase the sensitivity of cancerous tissue to radiation while reducing toxicity for healthy tissues. This development could improve the effectiveness of radiotherapy and extend the life of cancer patients [10].

## 3. Radiosensitizers

**Figure 2:**
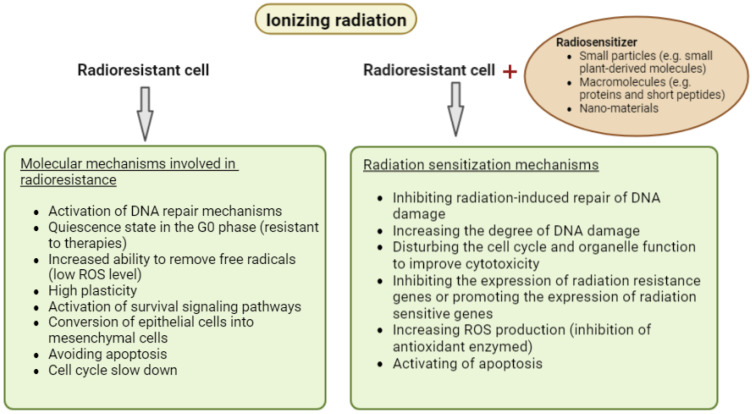
Molecular mechanisms involved in cancer cells’ radioresistance and the mechanism of radiosensitizer action.

Radiosensitizers are chemical compounds designed to increase the sensitivity of tumors to ionizing radiation and promote tumor inactivation more than using only one of the factors. The postulated mechanisms of action for radiosensitizers have changed along with the development of research and continuous technological innovations that allow the introduction of new materials and drugs to enhance the effects of radiotherapy [10,24,25]. The main mechanisms of action for these compounds are now assumed to be: (I) Inhibiting radiation-induced repair of DNA damage, increasing the degree of DNA damage; (II) disturbing the cell cycle and organelle function to improve cytotoxicity; and (III) inhibiting the expression of radiation resistance genes, or promoting the expression of radiation-sensitive genes (Figure 2) [10]. Recent research has divided radiosensitizers into three categories based on their structures: small particles (oxygen and oxygen mimetics, small plant-derived molecules, hypoxia-specific cytotoxins), macromolecules (proteins and short peptides, miRNAs, siRNAs, oligonucleotides), and nano-materials (metallic and nonmetallic nano-materials) [10]. The effective action of radiosensitizers has so far been well confirmed in animal models. Many studies have shown that the increase of radiosensitivity of cancer cells under the influence of, inter alia, plant compounds, and hypoxia inhibitors, improves the effectiveness of radiotherapy and prolongs the life span of animals [26,27,28,29,30,31,32,33]. Unfortunately, there are still no results of clinical trials that would allow the clinical use of some radiosensitizers.

**Figure 3:**
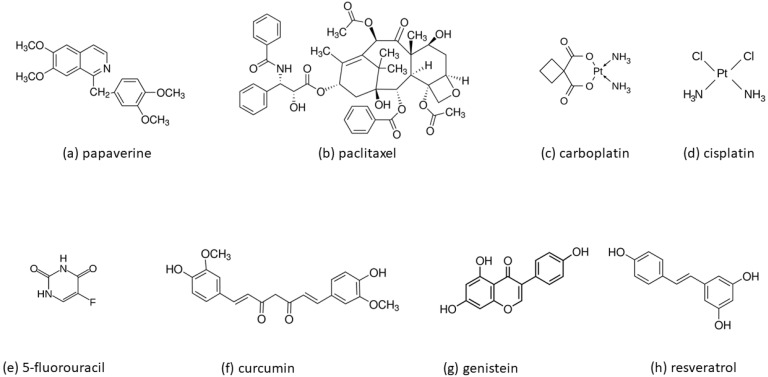
Molecular structures of small plant-derived molecules radiosensitizers discussed in this review: (**a**) papaverine, (**b**) paclitaxel, (**c**) carboplatin, (**d**) cisplatin, (**e**) 5-fluorouracil, (**f**) curcumin, (**g**) genistein and (**h**) resveratrol.

In recent years, more researchers have reported that small plant-derived molecules, often called “active compounds from Chinese herbs”, may enhance the sensitivity of cancer cells to radiotherapy [34]. In this review article, we only discuss some of the naturally occurring radiosensitizers currently in clinical trials (paclitaxel, curcumin, genistein, papaverine) (Table 1) and resveratrol, the sensitizing effect of which has been repeatedly confirmed in many independent studies. The molecular structures of all compounds discussed in this paper are shown in Figure 3.

**Papaverine** is an FDA-approved opium non-narcotic alkaloid used as a smooth muscle relaxant for treating vasospasm and erectile dysfunction. Its vascular activity is due to its role as a phosphodiesterase 10A inhibitor [35]. Benej et al. found that papaverine is a dual-activity drug that reversibly inhibits mitochondrial complex I [13]. In vivo, papaverine increases model tumor oxygenation at FDA-approved doses within 30 min. A single dose of the drug prior to radiotherapy alleviates hypoxia and significantly enhances the RT response. Most importantly, papaverine only requires a single dose 45 min prior to irradiation for radiosensitization. The drug is rapidly eliminated, so dosing can be repeated daily during hypofractionated radiotherapy [36]. Papaverine, as a radiosensitizer, combined with Stereotactic Body Radiotherapy (SBRT), is being evaluated in a Phase I clinical trial (NCT03824327) for the treatment of non-small cell lung cancer (NSCLC) or lung metastases. Another clinical study (NCT05136846) in which papaverine in combination with radiotherapy, chemotherapy, and immunotherapy is used in patients with unresectable locally advanced NSCLC was started in late 2021. In both cases, these are new studies, hence the lack of results.

**Paclitaxel** is an organic chemical compound belonging to the group of taxanes. For the first time, it was isolated from the bark of short-leaved yew (*Taxus brevifolia*). Paclitaxel is a cancer medicine used in chemotherapy. For treatment, it was introduced in 1992 as a preparation called Taxol [37]. Paclitaxel leads to the inhibition of cell division. It can bind to proteins involved in the formation of microtubules, favoring their formation, and then stabilizes them so that the breakdown of microtubules is inhibited. The action blocks the mitosis process in the G2/M phase and stops the proliferation of cancer cells, sensitizing them to the effects of ionizing radiation [38]. Paclitaxel and its modifications, e.g., nab-paclitaxel (albumin-bound paclitaxel), are widely used clinically for the treatment of many types of cancer. Many clinical trials concern the use of these compounds with other chemotherapeutic agents (e.g., carboplatin, cisplatin, gemcitabine), radiotherapy, immunotherapy (e.g., trastuzumab, pertuzumab), or a combination of several therapies as reported on ClinicalTrials.gov. Currently, about 200 clinical trials are registered in which paclitaxel is used in combination with radiotherapy (in many cases additionally with immunotherapy and with other drugs used in chemotherapy). In this review, we only cite a few examples of studies where paclitaxel was used only with radiotherapy (e.g., NCT02280252), with radiotherapy and immunotherapy (NCT00238420), as well as with radiotherapy and other chemotherapeutic agents (e.g., NCT01591135).

One of the clinical trials in which paclitaxel is used concomitantly with radiotherapy includes patients with locally advanced breast cancer (NCT02280252), as well as patients with stage II and III breast cancer (NCT00006256). These studies are currently in Phase II and the results have not been published so far.

Other clinical studies on paclitaxel have included a three-arm randomized Phase III trial (NCT02459457) conducted on a group of people with inoperable advanced esophageal squamous cell carcinoma (ESCC), in which the effects of three paclitaxel combinations were compared: paclitaxel—cisplatin, paclitaxel—carboplatin and paclitaxel—5-fluorouracil with radiotherapy and a study of paclitaxel with 5-fluorouracil in locally treating advanced esophageal cancer (NCT01591135) in Phase III. In the first of these studies (NCT02459457), none of the combinations of paclitaxel, i.e., with fluorouracil, cisplatin, or carboplatin, showed a significantly better overall survival in ESCC patients. In addition, studies have shown higher rates of hematological and gastrointestinal toxicity in the cisplatin group compared to the fluorouracil or carboplatin group [39]. The second clinical trial (NCT01591135) assessed the efficacy and safety of the paclitaxel plus fluorouracil regimen compared to the cisplatin plus fluorouracil regimen in combination with radiotherapy. The results obtained indicate that the paclitaxel plus fluorouracil regimen did not significantly extend the overall survival compared to the standard cisplatin plus fluorouracil regimen [40]. Other clinical trials with paclitaxel and carboplatin in combination with radiotherapy were performed in patients with serous papillary uterine carcinoma (an aggressive variant of endometrial cancer) (NCT00231868). Research has shown that radiotherapy “sandwiched” between paclitaxel/carboplatin chemotherapy is highly effective in women with completely resected papillary uterine cancer [41]. The research is currently in Phase II, as reported on ClinicalTrials.gov.

Paclitaxel in combination with radiotherapy and the monoclonal antibody (trastuzumab) has also been used in patients who underwent surgery for bladder cancer (NCT00238420). Clinical trials will evaluate whether administering paclitaxel together with radiation therapy and trastuzumab can kill more cancer cells. The studies are in phase I/II.

Radiotherapy and paclitaxel were also used in treating patients with nonmetastatic, unresectable pancreatic cancer (NCT00003591) [42]. The obtained data form the basis of new clinical trials that investigate paclitaxel and radiation in combination with a second radiosensitizer, gemcitabine (e.g., NCT02481635).

**Curcumin** is a natural polyphenolic compound that derives from the turmeric rhizome, also known as turmeric, which belongs to the ginger family. Initially, this plant was cultivated in Asia. Due to its wide range of biological activity, anti-inflammatory, and antioxidant properties, curcumin has been used as a natural medicine in traditional Chinese medicine for centuries. It can inhibit the transcription factor NFκB, which plays an important role in tumor formation and the resistance of tumor cells to IR [43]. Chendil et al. showed that the survival of PC-3 prostate adenoma cells treated simultaneously with ionizing radiation and curcumin decreased threefold, and the mechanism was related to NFκB [44]. Sandur et al. also demonstrated similar curcumin effects and radiation on HT29 and HCT116 human colon cancer cells. In addition to increased cell sensitivity to radiation due to the inhibition of the NFκB factor, NFκB-dependent antipoptotic (IAP2, Bcl-2, Bcl-XL), proliferative (cyclin D1), inflammatory (COX-2), and angiogenic (VEGF) genes target the expression of NFκB target genes [45]. Qian et al. examined the U87 human glioma cell line and proved that cell viability reduces due to curcumin and radiation. The cell cycle is stopped in the G2/M phase, causing this reduction. Moreover, the regulators of tumor progression: JNK and ERK map kinases, are inhibited [46]. In their studies on the normal MCF10A breast epithelial line and the MCF7 and MDA-MB-231, Minafra et al. treated breast tumor lines by combining radiation and curcumin. Their results showed that curcumin protects against IR in normal cells and reduces the survival rate of cancer cells that affect stimulation autophagy [47].

Many studies confirming the radiosensitizing properties of curcumin contributed to the fact that its activity has been assessed in clinical trials, including prostate cancer treatment [48]. Prostate cancer is the second most common cancer among the male population worldwide. Radiotherapy along with surgery is the basic method of treating patients with this type of cancer. Unfortunately, prostate cancer cells are only modestly responsive or even unresponsive to the cytotoxic effects of radiotherapy. The effect of curcumin as a potential radiosensitizer has been tested in patients with prostate cancer in clinical trials NCT01917890. It was shown that curcumin intake significantly increased total antioxidant capacity, with a reduction in SOD activity. Prostate-specific antigen (PSA) level was lowered in both the groups (curcumin or placebo group), but there was no significant difference in treatment outcomes between the groups [49].

A factor that has still limited the clinical value of curcumin is its low bioavailability. However, modern strategies have been developed that aim to increase the bioavailability of curcumin (e.g., nanocurcumin, liposomal form) [48]. The effect of nanocurcumin as a potential radiosensitizer has also been tested in patients with prostate cancer (NCT02724618). This was the first clinical experience of the use of nanocurcumin in prostate cancer patients. Saadipoor et al. showed that this randomized, controlled study did not demonstrate the efficacy of nanocurcumin for concurrent therapy in prostate cancer patients undergoing radiotherapy [50].

Curcumin has also been used in clinical trials before surgery in treating patients with rectal cancer (NCT00745134). The purpose of these tests is to find out whether giving chemotherapy (capecitabine) with radiation before surgery can shrink the tumor and reduce the amount of normal tissue that needs to be removed. The study will also evaluate whether chemotherapy and radiation therapy are more effective with or without curcu-min when given before surgery in patients with rectal cancer. The research is in phase II. The estimated date of completion of the study is March 2023.

Another clinical trial (NCT04294836) with curcumin is treating patients with advanced cervical cancer. In studies, curcumin is administered orally and chemotherapy (cisplatin) plus simultaneous radiotherapy (teletherapy + high- or low-frequency brachytherapy) is used. This research is currently in phase II. The estimated date of completion of the study is December 2023.

**Genistein** is a natural compound from the group of isoflavones, first isolated from the gorse. Genistein has anti-angiogenic and anti-tumor effects. In an in vitro study, Shi combined genistein treatment with irradiation of cervical cancer cells (Hela). His studies showed increased cell sensitivity to radiation by lowering survivin, which inhibited caspase 9, leading to apoptosis blockade [51]. Liu et al. conducted research on MCF7 and MDA-MB-231 breast cancer cells. The cells were treated with genistein and ionizing radiation. Scientists have shown an increase in DNA damage, cell cycle arrest (G2/M), and the enhancement of radiosensitivity through the apoptotic pathway [52]. The combined effects of ionizing radiation and genistein on A549 non-small cell lung cancer cells were investigated by Liu et al. Scientists have shown that combination therapy has led to an increase in the level of apoptosis and A549 cells’ increased sensitivity to radiation [53]. Genistein has been used in clinical trials in locally advanced squamous cell carcinoma of the head and neck patients to sensitize cancer cells and decrease side effects caused by radiotherapy and cisplatin (NCT02075112). Clinical research is in Phase I and there is no data on the results obtained. The effect of genistein as a potential radiosensitizer has also been tested in patients with bone metastases (NCT00769990). These studies are currently in Phase I or II and there is also a lack of research results.

## 4. Resveratrol

Resveratrol (3,4′,5-trihydroxystilbene, Figure 3h) is the best known and best-characterized derivative of stilbene due to its diverse potent biological activities and medicinal properties. Several excellent review articles provide a wealth of knowledge on the antioxidant, anti-inflammatory, cardioprotective, neuroprotective, and antitumor actions of resveratrol [54,55,56,57,58]. It is present in several dietary sources, including grapes (*Vitis vinifera*), soybeans, blueberries, pomegranates, and peanuts. Despite the significant biological potential of resveratrol, its use in medical therapies is still limited due to its low bioavailability. When ingested orally, stilbene derivatives are highly absorbed by the gastrointestinal tract, quickly and extensively metabolized in the liver, and then excreted through urine. Only a minor fraction of the absorbed compound will eventually reach the internal organs. Therefore, RSV’s low bioavailability must be overcome to be considered a therapeutic agent for enhancing the effects of radiotherapy. In this aspect, there is high hope for nanotechnology-based carriers. Nanoparticles have improved RSV solubility, stability, pharmacokinetics, and biodistribution in cancer tissues [59,60,61,62].

### 4.1. Resveratrol in Longevity and Cancer Therapy in Animal Model Studies

The health-promoting properties of resveratrol contributed to its recognition as a potential longevity drug. One of the main factors accelerating the aging process is excessive oxidative stress. Resveratrol improves the cells’ ability to maintain the ROS antioxidant balance. First, it has ROS scavenging properties [63]. Second, resveratrol enhances/maintains a high level of GSH and the activity of glutathione peroxidase (GPx), GSH transferase, superoxide dismutase (SOD), and catalase (CAT) [64,65]. The increased antioxidant enzyme activity may be stimulated by the influence of resveratrol on nuclear factor-erythroid-2 related factor 2 (Nrf2) [66]. The anti-aging effect of resveratrol is also related to its ability to improve mitochondrial function [67]. Wang and colleagues showed that RSV promoted mitochondrial function, up-regulating AMPK/SIRT1/Pgc1α, and down-regulated Akt/mTOR pathway activity in the zebrafish model [68]. Growing evidence confirms that low-grade chronic inflammation plays an important role in the progression of aging and age-related diseases [69]. Resveratrol also has a beneficial effect on these processes [54,55]. Based on mouse models studies, it was found that RSV promotes an increased activity of nuclear factor kappa B (NF-κB) and SIRT1, which results in the reduction of inflammatory markers such as interleukin-1β (IL-1β), tumor necrosis factor α (TNF-α) and monocyte chemoattractant protein-1 (MCP-1) [70,71]. Health and longevity are related to the effective maintenance of physiological, biochemical, and immune functions. To do this, the body creates new cells to replace old or damaged cells (e.g., cancer cells). Apoptosis is involved in this process. However, if apoptosis is dysregulated, age-related pathologies are likely to emerge. For example, excessive apoptosis of neurons promoted the development of neurodegenerative diseases [72,73]. Studies have shown that resveratrol treatment improved cognitive dysfunction by reducing neuronal apoptosis [74,75,76]. Molecular analysis showed that resveratrol increased the expression of SIRT1 and Bcl2, and decreased the expression of RhoA and cleaved caspase-3 [74,75].

Resveratrol, in addition to its well-documented properties in the prevention of age-related diseases and aging, has been extensively studied in terms of radiotherapy. However, in this case, resveratrol has a double face. On one hand, it can increase the radioresistance of cells, which is very beneficial in protecting healthy tissues during radiotherapy. On the other hand, many studies show that it can increase the sensitivity of cancer cells to radiation (literature review in a separate chapter below). The effectiveness of resveratrol in both processes depends on many factors, such as dose, tumor pattern, and animal species, as well as on other variables such as the sex and strain of the animals, method, and time of administration. A detailed review of the literature and a discussion of the topic can be found in several works [77,78,79,80,81]

Due to its strong anti-inflammatory and antioxidant properties, resveratrol is an effective radioprotector. The studies carried out so far allow us to identify pathways, the activation/inhibition of which increases the resistance of cells to IR. In vivo experiments showed that the radioprotective effects of resveratrol were associated with the amelioration of DNA damage [82,83]. A few studies indicated that it was a consequence of an increase in SIRT1 expression [84,85,86]. SIRT1 by stimulating p53 activity, overexpression of SOD2, and glutathione peroxidase 1 (GPX1) led to a reduction of apoptosis and amelioration of DNA damage [84,85]. The radioprotective effect of resveratrol was also confirmed by Said et al. [87] and Radwan et al. [88]. In this case, the action of resveratrol was associated with a reduction in the contents of inflammatory cytokines; TNF-α, nuclear factor-kappa (NF-κB), and interleukin 1β (IL-1β). Western blotting analysis revealed that resveratrol down-regulated the proteins expression of phosphoinositide 3-kinases (PI3K), protein kinase B (Akt) as well as the mammalian target of rapamycin (mTOR) [88].

In addition to the radioprotective effect of resveratrol, a synergistic effect in combination with ionizing radiation has been observed in many studies. The radiosensitizing effects of resveratrol are well documented in in vitro studies as discussed in detail in a separate section of this review. However, in just a few in vivo studies, the authors have confirmed that resveratrol can enhance radiotherapy. In one study, the radiosensitizing mechanism of resveratrol’s action is associated with increased autophagy and apoptosis [89]. Mikami et al. also showed that resveratrol can increase the efficacy of irradiation through the regenerating gene (REG) III expression pathway [90]. In other studies, the authors suggest that resveratrol in combination with ionizing radiation delayed repair of radiation-induced DNA double-strand break (DSB) and prolonged G2/M arrest, which induced apoptosis [91].

### 4.2. Clinical Trials of Resveratrol in the Treatment of Cancer and Selected Age-Related Diseases

Extensive research on resveratrol began when Renaud and de Logeril described an epidemiological study of the association between coronary heart disease and high red wine consumption in France. This phenomenon has been called the French Paradox [92]. A significant number of studies, including in vitro and preclinical studies, have shown that resveratrol is potent against chronic inflammatory diseases such as cancer and age-related diseases [93,94,95,96].

The effects of resveratrol in treating patients with various types of cancer (multiple myeloma, breast cancer, follicular lymphoma, and neuroendocrine tumors) have been investigated in several clinical trials. However, the majority of clinical trials are focused on evaluating the effects of resveratrol in colorectal cancer development. This is due to direct contact and prolonged exposure to colonic tissue. Moreover, the intestinal epithelium seems to be better adapted to the absorption of active molecules compared to other tissues. The oldest clinical study registered in the Clinic.trials.gov database dates back to 2005 (NCT00256334). The aim of the study was to determine the effect of freeze-dried grape powder (GP) on the Wnt signaling pathway in patients with colorectal cancer. Studies have shown that low doses of resveratrol in combination with other bioactive ingredients decreased the expression of the *Wnt* gene within the normal mucosa, but had no effect on the cancerous mucosa. This indicates that GP may play a beneficial role in the prevention of colon cancer, and not in the treatment of diagnosed colon cancer [97]. Other studies focusing on the same type of cancer, were carried out by Patel et al. [98]. They showed that administering resveratrol to patients with histologically confirmed colorectal cancer for 8 days at a dose of 0.5 or 1 g per day reduced the proliferation of cancer cells by 5%. The results suggest that a daily oral dose of 0.5 or 1.0 g of resveratrol is sufficient to induce anti-carcinogenic effects. The low systemic availability of resveratrol due to its rapid and intensive metabolism significantly limits its usefulness in the treatment of neoplasms located in organs distant from the absorption site. Howells and colleagues used micronized resveratrol (SRT501) in patients with colorectal cancer and liver metastases scheduled for hepatectomy (NCT00920803). They found that SRT501 was better tolerated by patients (3.6 times higher level compared to non-micronized resveratrol) and all side effects were considered mild compared to non-micronized resveratrol. A significant increase in the marker of apoptosis (cleaved caspase-3) was observed in tumor tissue compared to the tissue from patients receiving a placebo [99]. The results of clinical trials conducted in colorectal cancer patients indicate that resveratrol exhibits some pharmacological activity, it is unclear whether this effect is significant enough to be a useful therapeutic agent in the treatment of this type of cancer.SRT501 alone or in combination with bortezomib has been used in patients with relapsed and/or refractory multiple myeloma (NCT00920556). In this study, researchers noted adverse events such as nausea, diarrhea, vomiting, fatigue, anemia, infections, and most specifically renal failure. The main finding of the study was unexpected renal toxicity that led to the early termination of the study [100]. Despite the very promising in vitro studies described by Bhardwaj et al., [101], in which the combination of resveratrol and bortezomib achieved synergistic cytotoxicity in multiple myeloma cells, clinical studies do not support such an effect in humans.

Kjaer and colleagues conducted a randomized placebo-controlled clinical study using two doses of resveratrol (150 mg or 1000 mg resveratrol daily) for 4 months in 66 middle-aged men suffering from metabolic syndrome. The highest dose of resveratrol significantly lowered the serum levels of the androgen precursors androstenedione, dehydroepiandrosterone, and dehydroepiandrosterone sulfate. However, the prostate size and circulating levels of PSA, testosterone, free testosterone, and dihydrotestosterone were unchanged [102]. The authors do not support the use of resveratrol in the treatment of benign prostatic hyperplasia. In subsequent clinical trials the efficacy of a commercial preparation of powdered skin from muscadine grapes (containing ellagic acid, quercetin, and resveratrol) in patients with nonmetastatic biochemically recurrent prostate cancer has been evaluated (NCT01317199). In the first phase of the research, it was shown that the preparation, even at a high dose, 4000 mg/day, was safe for patients [103]. Then Paller and colleagues conducted a randomized, multicentre, placebo-controlled phase II clinical study [104]. A study carried out on 125 patients provided evidence that the polyphenol-rich muscadin grape skin extract did not benefit the overall prostate cancer patient population. Based on these studies, it seems unlikely that resveratrol will be an effective treatment for prostate cancer, but more clinical trials need to be conducted to confirm this.

Alternatively, in the case of breast cancer, one clinical trial has indicated that resveratrol may be a promising therapeutic agent for such diseases [105]. The results showed that total trans-resveratrol and glucuronide metabolite serum levels increased over time. In addition, the authors demonstrated the dose-dependent effects of resveratrol on methylation of *RASSF-1α*, a gene associated with breast cancer. *RASSF-1α* methylation decreased with increasing levels of trans-resveratrol and resveratrol-glucuronide in the circulation, and with decreasing breast-specific prostaglandin (PG)E2 expression in the breast. These results suggest that resveratrol may influence the epigenetics of the genes associated with breast cancer, which should be confirmed in future clinical trials. Other studies focusing on the same type of cancer are still ongoing with no results so far. One of the trials (NCT04266353) investigates the inhibitory effect of resveratrol on insulin-like growth factor II (IGF-II) expression in African American women with breast cancer. The purpose of the second study (NCT05306002) is to assess whether DNA damage in patients with breast and ovarian cancer syndrome decreases with dietary components, including resveratrol. Based on the presented clinical trials, it is difficult to definitively conclude about the therapeutic effectiveness of resveratrol, taking into account the small number of clinical trials conducted.

Numerous in vitro and in vivo studies have confirmed the beneficial effects of resveratrol in age-related diseases, e.g., such as neurodegenerative disease, diabetes, and atherosclerosis. The ClinicalTrials.gov database has registered 5 clinical studies on the effects of resveratrol on Alzheimer’s disease (AD), of which the results of 2 tests have been published (NCT00678431, NCT01504854). Both studies showed that RSV is safe and well tolerated in patients with mild to moderate AD [106,107,108]. Resveratrol and its major metabolites penetrated the blood-brain barrier and affected the cerebrospinal fluid. Compared with placebo-treated patients, patients treated with resveratrol had a slower decrease in beta-amyloid 42 (Aβ42) and Aβ40 in the cerebrospinal fluid, indicating less accumulation of Aβ in the brain. It was also observed that the levels of tau and phospho-tau in the cerebrospinal fluid remained unchanged. The authors concluded that targeting the molecular pathways of aging could lead to new therapies that will delay or prevent aging-related diseases, including AD. Both clinical studies presented results consistent with those obtained in in vitro and in vivo studies and provided evidence that resveratrol may be a safe and effective treatment for AD [109].

Diabetes mellitus (DM) is another common, chronic, and serious disease in which resveratrol may be effective. In clinical trials, Brasnyo et al. investigated whether resveratrol improves insulin sensitivity in patients with type 2 diabetes mellitus (T2DM). The levels of glucose-dependent insulinotropic polypeptides (GIP) and glucagon-like peptide 1 (GLP-1) (hormones affecting postprandial hyperglycemia) and the ratio of phosphorylated protein kinase B (pAkt) to protein kinase B (Akt) were also tested. In diabetic patients, resveratrol did not alter GLP-1 or GIP levels, but significantly reduced insulin resistance and blood glucose levels, and delayed postprandial glucose peaks. The authors suggested that resveratrol improved insulin sensitivity in humans by reducing oxidative stress which leads to more efficient insulin signaling through the Akt pathway [110]. Similarly, promising results in clinical trials (CTRI/2011/05/001731) were obtained by Bhat et al. [111]. A study revealed that daily resveratrol treatment for 3 months decreased glycated hemoglobin (HbA1c) levels, systolic blood pressure, total cholesterol, and total protein, improving glycemic control. The authors concluded that oral supplementation of resveratrol could be a potential adjuvant for the treatment of diabetes [111]. Thazath et al. drew the opposite conclusions about the use of resveratrol to improve glycemic control [ACTRN12613000717752]. The authors found that in T2DM patients, resveratrol had no effect on GLP-1 secretion, glycemic control, gastric emptying, body weight, or energy intake [112]. Subsequent clinical trials (NCT02565979, NCT02129595) by Ligt and colleagues also found that resveratrol supplementation did not increase insulin sensitivity [113,114].

Resveratrol is one of the most active polyphenols that stimulate SIRT-1 activity [115]. The activity of this protein is decreased in T2DM [116]. The clinical trials (NCT02244879) evaluated whether an increase in SIRT-1 expression/activation affected the acetylation of histone 3 at the 56th lysine residue (H3K56ac) in T2DM patients who were receiving either resveratrol or placebo for 6 months. Unexpectedly, it was found that not all patients receiving resveratrol displayed increased SIRT-1 expression/activation. Nevertheless, the increase in SIRT-1 expression was associated with significant H3K56ac content reduction and increased serum antioxidant activity in diabetes patients [117]. The same clinical studies demonstrated that 6-month supplementations with 500 or 40 mg/day of resveratrol increased pentraxin 3 (anti-inflammatory protein produced in response to inflammation [118]) level and total antioxidant status in type 2 diabetic patients [119]. Increased expression of SIRT1 and AMPK in skeletal muscle was confirmed in other clinical trials in diabetic patients supplemented with resveratrol (NCT01677611). These results indicated that resveratrol may have beneficial exercise-mimetic effects in type 2 diabetes patients [120]. Analysis of data from registered controlled trials by Fraiz et al. showed that RSV supplementation can stimulate SIRT-1 in humans and thus contribute to the treatment of overweight and its comorbidities. However, more research is needed as this effect has not been really confirmed [121]. 

A critical review of clinical trials evaluating the efficacy and safety of resveratrol preparations for adults with type 2 diabetes was conducted by a team of scientists from the University of Manitoba [122]. The authors conclude that the current studies are insufficient to assess the safety and efficacy of resveratrol supplementation in the treatment of adults with type 2 diabetes. The limited available studies do not provide sufficient evidence to support any beneficial or unfavorable effect of four to five weeks of resveratrol at a dose of 10 mg to 1000 mg in adults with type 2 diabetes [121].

Atherosclerosis is considered the major cause of cardiovascular diseases (CVD). It is characterized by the deposition of extracellular lipids, the proliferation and migration of local smooth muscle cells, and chronic inflammation. It leads to luminal narrowing and/or thrombus formation, resulting in clinical events such as coronary artery disease, peripheral arterial disease, or stroke [123,124]. Contrary to the promising results of animal studies, clinical trials evaluating the effect of resveratrol on the plasma lipid profile in humans have not been conclusive. For example, in clinical trials conducted in healthy obese men and healthy adult smokers treated for 30 days with resveratrol 150 mg/day and 500 mg/day, respectively, significant reductions in plasma triglycerides were found [125,126]. Another study in patients with type 2 diabetes revealed that supplementation of resveratrol for 3 months significantly improves total cholesterol. No significant changes in body weight and high-density lipoprotein and low-density lipoprotein cholesterols were observed [111]. Tome-Carneiro and colleagues showed that in statin-treated patients with high CVD risk, a daily intake of 350 mg of resveratrol-enriched grape extract containing 8 mg of resveratrol resulted in a 20% reduction in oxidized LDL cholesterol and a 4.5% reduction in LDL cholesterol [127]. However, many clinical studies have not confirmed the effect of resvertrol on the lipid profile in non-obese women with normal glucose tolerance [128], clinical trials NCT00823381), obese men (NCT01150955) [129], mildly hypertensive adults, overweight/obese adults [130] and in patients with the metabolic syndrome [131]. Similar conclusions from systematic review and meta-analysis on randomized clinical trials were made by Haghighatdoost and Hariri [132]. The results indicated that resveratrol supplementation did not change circulating low-density lipoprotein, total cholesterol, and high-density lipoprotein, while triacylglycerol showed significant increases after taking resveratrol supplements. The authors conclude that resveratrol does not change lipid profile concentration [132].

A clinical trial has also been conducted on the effects of resveratrol on the primary prevention of atherosclerosis (NCT01244360). The clinical trial focused primarily on endothelial responses and plasma biomarkers in healthy subjects. Changes in the expression of genes associated with inflammation and atherosclerosis were assessed, including interleukins (IL), interferon-gamma (IFN-γ), TNF-α, a vascular cell adhesion molecule (VCAM), and an intercellular adhesion molecule (ICAM). Treatment with resveratrol has been shown to reduce the expression of intercellular adhesion molecules (ICAM), vascular cell adhesion molecules (VCAM), and interleukin-8 molecules that contribute to the development of atherosclerosis by promoting lipid deposition and exacerbating inflammation [133]. Overall, more clinical data is needed to fully understand the therapeutic potential of resveratrol. Until then, resveratrol will continue to be promising, but not yet proven.

### 4.3. Resveratrol as a Radiosensitizer

In light of mechanisms favoring and limiting the effectiveness of radiotherapy, resveratrol action may appear contrary to the desired properties of substances by increasing the sensitivity of cells to ionizing radiation. However, the effect of resveratrol on cells follows the classic concept of hormesis, i.e., the theory that the dose of a given factor determines whether it will have a beneficial or harmful effect on the organism. All the aforementioned effects related to resveratrol consumption through food products mainly result from its consumption in relatively small amounts. With an appropriately high dose, resveratrol can act in a manner opposite to the standard antioxidant effects of polyphenols. This is especially important in the context of sensitization of neoplastic cells to ionizing radiation, which, depending on the type of neoplasm, exhibit a number of mechanisms that make them more resistant to radiation.

One such mechanism is the increased expression of STAT (signal-transducer-and-activator-of-transcription) transcription factors, which are overexpressed in the head and neck squamous cell carcinoma (HNSCC). This condition promotes the growth and survival of cancer cells. The phosphorylated form of STAT3 reduced the level of the p53 tumor suppressor, increased the activity of anti-apoptotic proteins, and increased the tumor’s resistance to chemotherapeutic agents and ionizing radiation. These processes are triggered by the stimulation of vascular endothelial growth factor (VEGF), matrix metalloproteinases-2 and -9 (MMP-2 and MMP-9), and inhibitors of apoptosis protein-1 (IAP-1) [134,135]. In FaDu cells, resveratrol could significantly lower the level of STAT3 phosphorylation by activating the suppressor of cytokine signaling 1 (SOCS-1), an inhibitor of STAT3. As a result, cell proliferation was inhibited, apoptosis induced, and the use of 100 µM resveratrol increased cell sensitivity to radiation at a dose of 10 Gy [136]. Yang et al. [137] had similar observations on radioresistant glioblastoma multiforme tumor-initiating cells (GBM-TIC). In their study, cells were irradiated with doses of radiation at varying intensities, which increased STAT3 phosphorylation, and GBM-CD133^+^TIC cells were characterized by high tumorigenic capacity and radiochemical resistance. The use of 100 µM resveratrol increased the cell sensitivity to radiation and induced apoptosis by inhibiting STAT3 signaling. Apart from the induction of apoptosis, resveratrol may also induce cell cycle inhibition, autophagy and reduce the response of cells to DNA damage, leading to further radiosensitization [90,138].

The group of neoplasms in which radiotherapy is the first-line treatment strategy is prostate neoplasms. Despite this, prostate cancers (PC) become resistant to this type of treatment. Among the factors influencing the emergence of tumors, radiation resistance is the inhibition of DOC-2/DAB2 (DAB2IP) protein activity. This protein participates in regulating cell proliferation, cell survival, and apoptosis by inactivating the phosphatidylinositol 3-kinase/Akt signaling pathway. During the study on the human DAB2IP-deficient prostate cancer cells resistant to IR (LAPC4-KD and PC3-KD cell lines), irradiation with X-rays in doses of 2–6 Gy was used after prior incubation of cells with 25 or 500 µg/mL of resveratrol. Resveratrol could enhance the effect of irradiation by inhibiting cell proliferation, inducing apoptosis, arresting the cell cycle, and delaying and slowing down cellular responses to radiation-induced DNA damage [91].

Another mechanism that sensitizes prostate cancer cells to ionizing radiation is the activity of the perforin and granzyme B proteins, which are an important element of the killer properties of lymphocytes and NK cells. Fang et al. showed that resveratrol up-regulated the perforin and granzyme B expression in prostate cancer cells (line PC-3 and DU145). Ionizing radiation alone up-regulated the expression of granzyme B, but not that of perforin. The combined IR and resveratrol treatment of PC-3 and DU145 cells increased the expression of both perforin and granzyme B. A significant reduction was observed in survival and a remarkable increase in apoptosis [139]. The same authors in subsequent studies confirmed that RSV synergizes with IR to inhibit the proliferation of a PC cell line by promoting apoptosis and senescence. The antiproliferative effect of RSV/IR correlated with increased expression of antiproliferative molecules p15, p21, and mutant p53 and decreased expression of cyclin B, cyclin D, and cell division protein kinase 2 (cdk2). Increases in apoptosis correlated with the increased expression of pro-apoptotic molecules such as the Fas receptor and TRAIL receptor 1 (TRAILR1). Similar to apoptosis, cellular senescence has been shown to function as a potent mechanism to inhibit tumor cell proliferation and survival; phosphorylation of histon H2A.X has been suggested as a marker for cell senescence. Fang et al. showed that RSV/IR promoted senescence as evidenced by the increased expression of H2A.X [140].

Rashid et al. also confirmed resveratrol’s high efficiency in sensitizing prostate cancer cells to ionizing radiation [141]. The authors showed that resveratrol concentrations similar to those achieved in human serum (2.5 and 5 μM) enhanced the cytotoxicity of a conventional radiation therapy fraction (2 Gy) in PC cells without additional toxicity to normal epithelial cells. They performed studies proposing resveratrol’s model of action and ionizing radiation in regulating the cell cycle and survival in PC cells. Accordingly, RSV enhances the effects of IR on kinase AMPK activation, leading to an early cell cycle arrest, and inhibits Akt kinase to reduce gene expression, proliferation, and enhance radiation cytotoxicity [141].

The synergistic effect of resveratrol and radiation on inhibiting the cell survival of radioresistant PC cells (exactly DU145 cells) was confirmed in another study. Scarlatti et al. showed that resveratrol potentiates ionizing radiation-induced ceramide accumulation by promoting its de novo biosynthesis [142]. Ceramide accumulation is an important pro-apoptotic signal enabled by the activation of sphingomyelinases as a response to various stress factors (i.e., tumor necrosis factor). Ceramide activates a serine-threonine protein phosphatase, and in cells, it regulates protein phosphorylation as well as multiple downstream targets (such as interleukin converting enzyme (ICE)-like proteases, stress-activated protein kinases, and the retinoblastoma gene product) that mediate its distinct cellular effects [143].

Melanomas are the most aggressive skin cancer type, which is also characterized by high resistance to chemotherapeutic agents. Radiotherapy is used as an adjuvant treatment after surgery and alongside chemotherapy, mainly to control metastasis. The 50 µM resveratrol concentration combined with a 5 Gy radiation dose resulted in the reduced survival of SW1 and WM35 melanoma cells [144]. Studies on cultured mouse melanoma B16F10 and mouse colon carcinoma CT26 cells showed that resveratrol led to the radiosensitization of cancer cells by increasing apoptotic cell death and loss of mitochondrial membrane potential, presumably through enhanced ROS generation [145].

The use of resveratrol in the amount of 20 µM also enhances the sensitivity of non-small cell lung cancer cells at radiation doses from 0 to 8 Gy. This synergistic strategy induced radiosensitization through apoptosis-independent molecular pathways and increased the production of reactive oxygen species and DNA double-strand breaks, thus leading to accelerated cell aging and death [146]. Another study on A549 lung cancer cells proved that resveratrol could potentiate the effects of cell irradiation by limiting the cancer cell’s ability to regulate intracellular calcium concentration through the store-operated calcium entry (SOCE) mechanism. This result was achieved by reducing the expression of the key elements for this mechanism, i.e., the matrix stromal interaction molecule (STIM1) and a calcium release-activated calcium channel protein (Orai1) responsible for detecting the decrease in the concentration of calcium ions in the endoplasmic reticulum and triggering the influx of calcium ions from the extracellular space. This process can lead to the autophagy of cells [147]. This mechanism has also been identified earlier in prostate, colorectal, and melanoma cancer cells [148,149].

Another mechanism that may be influenced by resveratrol is the sirtuin 1protein (SIRT1). This protein can silence genes related to aging processes, and under normal conditions, it protects healthy cells against ionizing radiation. SIRT1 is apparently bifunctional in tumors and has both suppressor and oncogenic functions. There are reports that SIRT1 stimulation can induce cell proliferation, angiogenesis, and cancer cell resistance to treatment. However, these effects may differ in different types of neoplastic cells [150]. It has also been suggested that stimulating SIRT1 by resveratrol may regulate the amount of cyclin D1, leading to the inhibition of tumor cell proliferation and survival [151]. Research into this aspect of the response to resveratrol is fairly limited, and its combination with ionizing radiation has different ramifications for different cell types. An analysis of resveratrol and IR effects on A549 and H460 lung cancer cells showed that SIRT1 expression was negatively correlated with radiosensitivity in lung cancer cell lines. Radiation sensitivity was significantly reduced when SIRT1 was activated by resveratrol. The SIRT1 action was mainly related to the regulation of DNA damage repair and apoptosis processes (SIRT1/NF-κB/Smac pathway) [152]. Based on published findings, the SIRT1/NF-κB/Smac pathway may be a new potential target pathway in therapy for non-small cell lung carcinoma. Resveratrol combined with ionizing radiation appears to have the opposite effect, i.e., reducing the radiosensitivity of lung cancer cells.

Radiotherapy is a treatment method commonly used to treat glioblastoma. Standard treatment strategies include surgery followed by adjuvant radiochemotherapy. In this respect, resveratrol as a factor sensitizing cancer cells to radiation doses may increase the effectiveness of treatment [139,140]. Resveratrol was tested on SU-2 cells exposed to X-ray radiation in single doses from 0 to 6 Gy. The initial application of 75 µM resveratrol reduced the proliferation of cancer cells compared with radiation-only cells. There was also an increase in the microtubule-associated protein light chain 3 expression, which plays a key role in cell autophagy, and a reduction in the amount of anti-apoptotic protein Bcl-2 [90]. The BAX/Bcl-2 ratio was also reduced in the pituitary cancer model for GH3 and TtT/GF cells. Irradiation of cells after applying 10 µM resveratrol induced cell death, whereas the irradiation itself only limited their growth [153]. In another glioblastoma model, the inhibitory effect of resveratrol on the HIF-1α protein was observed on U87 MG cells. HIF proteins are elements of the cell’s response to the state of hypoxia. Their activation is associated with reducing apoptosis, cell differentiation, activation of DNA repair mechanisms, and stimulation of the formation of new blood vessels. All these effects are associated with tumor resistance to treatment, and the state of tissue hypoxia alone is a poor prognosis for the further course of the disease [154,155,156,157]. Hypoxic neoplastic cells also become resistant to ionizing radiation [158,159]. The use of resveratrol at a concentration of 20 µM before the combined action of 1 µM iododeoxyuridine (a substance that causes cell sensitization to radiation) and irradiation with a dose of 2 Gy reduced the ability of cells to form colonies. It increased the degree of DNA damage compared with cells treated only with iododeoxyuridine [160].

Radioresistance is also a hallmark of nasopharyngeal carcinomas (NPCs). Tumors belonging to this group are more common in the Asian continent and are often diagnosed in the advanced and metastatic stages. In order to overcome the radiation resistance of human CNE-1 cells, they were incubated with resveratrol at concentrations ranging from 25 to 150 µM prior to irradiation with X-rays (0–6 Gy). Resveratrol increased the cells’ sensitivity to radiation but decreased their viability and ability to form dose-dependent colonies. In this case, RSV’s radiosensitizing activity was based on the phosphorylated form of AKT inhibitors by reducing the level of transcription factor E2F1. Moreover, administering resveratrol in the NPC tumor xenograft models at 50 mg/kg/day and 4 Gy/day irradiation doses for the next 3 days significantly reduced the tumor volume and weight of treated mice compared with those exposed to radiation only or who were administered resveratrol alone [161].

The ability to sensitize cells to ionizing radiation was also studied in a breast cancer cell model. In the MCF-7 cell line, the combined action of resveratrol at various concentrations (0, 10, 30, 100 µM) combined with radiation at doses of 1, 2, and 3 Gy, induced cytotoxic effects, limited cell proliferation, and inhibited the cell cycle. Interestingly, the best results were achieved using 30 µM resveratrol combined with 3 Gy of radiation [162]. In subsequent studies conducted on the same cell model (MCF-7), it was shown that out of the selected stilbene derivatives (resveratrol, piceid, and piceatannol), resveratrol increased the effect of IR the strongest. Our results showed that RSV combined with IR led to a decrease in the activity of antioxidant enzymes, resulting in the accumulation of the formed ROS. RSV combined with IR reduced the activity of antioxidant enzymes, resulting in the accumulation of nascent ROS. The effects of resveratrol and IR also enhanced the expression of apoptotic genes, such as Bax, p53, and caspase 8, leading to apoptosis [163]. Amini et al. showed a similar sensitizing effect of resveratrol combined with radiotherapy in MCF-7 cells [164].

## 5. Summary

In vitro and in vivo data indicate that plant compounds can be effective in increasing the radiation sensitivity of cancer cells. Nevertheless, clinical applications of natural products in radiotherapy are scarce, which may be related to their low bioavailability in the human body. A growing body of research gives hope that the bioavailability and efficacy of radiosensitization can be significantly increased by using new drug delivery systems. However, only the well-documented biological activity of compounds is the basis for further research into methods to optimize bioavailability in humans.

This review presents clinical trials of natural radiosensitizers and discusses their mechanisms of action. If the clinical trials have been completed, the article also discusses their results. In addition, the review summarizes the huge number of preclinical studies investigating the radiosensitizing effects of resveratrol.

## Figures and Tables

**Table 1:** Registered Current Clinical Trials of small plant-derived molecules radiosensitizers (https://Clinicaltrials.gov/, accessed on 4 August 2022).

Identifier	Natural Compound	Tumor Type	No. of Patients	Phase of Clinical Trials	Drug Used in Combination with Radiotherapy
NCT03824327	Papaverine	Non-Small Cell Lung Cancer or Lung Metastases	24	Phase I	Papaverine
NCT05136846	Papaverine and Paclitaxel	Locally Advanced Non-Small Cell Lung Cancer	28	Phase I	Carboplatin + Paclitaxel + Durvalumab and Papaverine
NCT02459457	Paclitaxel	Local Advanced Esophageal Cancer	321	Phase III	Group 1: Paclitaxel + Cisplatin Group 2: Paclitaxel + Fluorouracil Group 3: Paclitaxel + Carboplatin
NCT01591135	Paclitaxel	Locally Advanced Esophageal Carcinoma	436	Phase III	Group 1: Paclitaxel + 5-fluorouracil Group 2: Cisplatin + 5-fluorouracil
NCT02280252	Paclitaxel	Breast Cancer	69	Phase II	Paclitaxel
NCT00238420	Paclitaxel	Bladder Cancer	70	Phase I/II	Paclitaxel + Trastuzumab
NCT00231868	Paclitaxel	Uterine Cancer	81	Phase II	Carboplatin + Paclitaxel
NCT00003591	Paclitaxel	Pancreatic Cancer	122	Phase II	Paclitaxel
NCT02724618	Curcumin	Prostate Cancer	64	Phase II	Nanocurcumin
NCT01917890	Curcumin	Prostate Cancer	40	Completed	Curcumin
NCT00745134	Curcumin	Rectal Cancer	45	Phase II	Capecitabine + Curcumin
NCT04294836	Curcumin	Cervical Cancer	240	Phase II	Cisplatin + Curcumin
NCT02075112	Genistein	Advanced Squamous Cell Carcinoma of the Head and Neck	24	Phase I	Soy isoflavone + Cisplatin
NCT00769990	Genistein	Bone Metastases	0	Phase I/II	Genistein

## Data Availability

Not applicable.
